# Supplementary material for: Aberrant Expression of RAD52, Its Prognostic Impact in Rectal Cancer and Association with Poor Survival of Patients
Source: Int J Mol Sci. 2020 Mar 4;21(5):1768. doi: 10.3390/ijms21051768 (PMC7084626; doi:10.3390/ijms21051768)
Supplement: Supplementary file 1 [file ijms-21-01768-s001.pdf]

Supplementary Materials: Aberrant expression of RAD52 associated with poor survival of patients and its prognostic impact in rectal cancer

Vincent Ho, Liping Chung, Amandeep Singh, Vivienne Lea, Askar Abubakar, Stephanie H Lim, Wei Chua, Weng Ng, Mark Lee, Tara Roberts, Paul de Souza, and Cheok Soon Lee

Supplementary Table S1 Treatment received by patients with rectal cancer

	Preoperative			Postoperative		Pre/Post-operative		Surgery alone	Unknown
	Neo RT	Neo Chemo	Neo Chemo-RT	Adj. Chemo	Adj. Chemo-RT	Neo Chemo-RT + Adj. Chemo	Neo RT + Adj. Chemo		
**n**	40	22	20	34	3	11	6	65	24

*Available study subjects (n=179) at the Liverpool Hospital. Neo, neoadjuvant; Adj., adjuvant; RT, radiotherapy; Chemo, chemotherapy

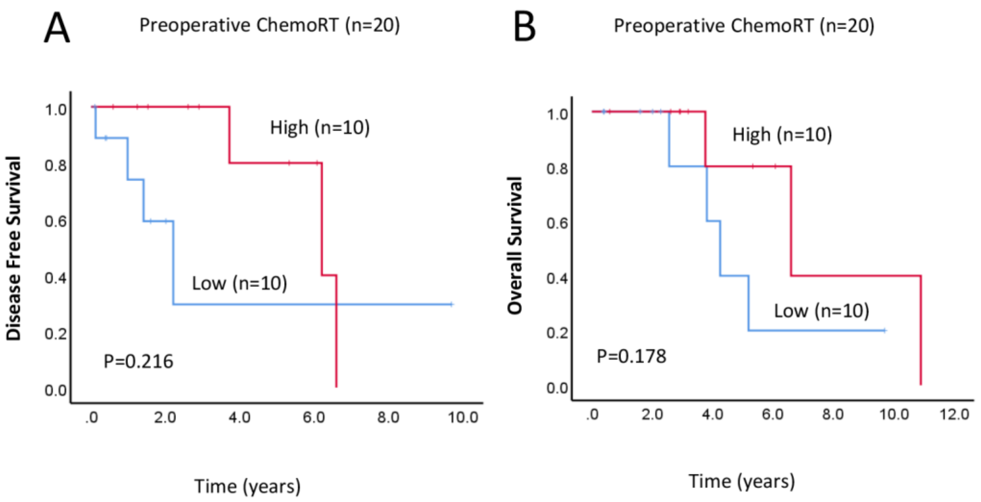

Supplementary Figure S1. Kaplan–Meier survival analysis in patients treated with chemo-radiotherapy. Relationship between RAD52 expression and disease-free survival (A) and overall survival (B) in chemo-radiotherapy treated rectal cancer patients.
